# Data for “Social-evaluative threat: Stress response stages and influences of biological sex and neuroticism”

**DOI:** 10.1016/j.dib.2019.104645

**Published:** 2019-10-13

**Authors:** Eefje S. Poppelaars, Johannes Klackl, Belinda Pletzer, Frank H. Wilhelm, Eva Jonas

**Affiliations:** University of Salzburg, Hellbrunnerstraße 34, 5020, Salzburg, Austria

**Keywords:** Social-evaluative threat, Gender, Cortisol, Anxiety

## Abstract

This Data In Brief article contains supplementary materials to the article “Social-evaluative threat: stress response stages and influences of biological sex and neuroticism” [1], and describes analysis results of an open dataset [2].

Additional information is provided regarding the methods, particularly: the analysis of individual stress response peak times per stress system, and the statistical analysis. Importantly, correlation tables are presented between the different stress systems, both for baseline stress levels as well as for stress responses, and significant associations are displayed in scatter plots.

**Table:** Specifications Table

Subject area	Psychology
More specific subject area	Neuropsychology and Physiological Psychology; Experimental and Cognitive Psychology.
Type of data	Table, Figure, text.
How data was acquired	Cardiovascular physiology (electrocardiography and impedance cardiography) and respiration were recorded continuously. Blood pressure, endocrine physiology, and self-reported states were repeatedly measured. Additionally, self-reported traits were assed via questionnaires at the end of the experiment.
Data format	Raw and analyzed
Experimental factors	Male and female participants were 18–35 years of age, right-handed, had normal or corrected- to-normal vision, were currently studying at college or university, were heterosexual, free of psychiatric and endocrinological disorders, not taking medication that could influence cognition, emotion, or hormones, and were not a regular smoker or drinker. Additionally, female participants did not use oral hormonal contraception or an intrauterine device for at least the last three months, were not currently pregnant or breast-feeding, had a regular menstrual cycle, and were tested during the luteal phase of their menstrual cycle.
Experimental features	A five-minute resting state was measured as a baseline. To induce social-evaluative threat (SET), an impromptu speaking task was used. Participants were first told in the lab that they would give a five-minute speech about their positive and negative personality characteristics. We told participants that their video would later be evaluated by that same audience on ten aspects concerning speech delivery, content, and quality. Participants were given five minutes to prepare their speech (stress condition). During the speech, the video of the neutral pre-recorded audience was shown while a camera recorded their speech. The entire SET manipulation lasted about 18 min. After the speech, a five-minute recovery was measured, and a second recovery 30 minutes later.
Data source location	Salzburg University, Salzburg, Austria
Data accessibility	E.S. Poppelaars, J. Klackl, B. Pletzer, F.H. Wilhelm, E. Jonas, Open dataset for: “Social-evaluative threat: Stress response stages and influences of biological sex and neuroticism”, *Mendeley Data*. (2019). https://doi.org/10.17632/7vj8r76s6f.
Related research article	E.S. Poppelaars, J. Klackl, B. Pletzer, F.H. Wilhelm, E. Jonas, Social-evaluative threat: Stress response stages and influences of biological sex and neuroticism, *Psychoneuroendocrinology. 109* (2019) 104378. https://doi.org/10.1016/j.psyneuen.2019.104378.

**Table:** 

**Value of the Data** − The correlation coefficients could be used in a meta-analysis about associations between stress responses. − The information about the timing of individual stress responses in different systems and their sex differences could inform research on the timing of stress response. − Our approach to missing data management ‒ particularly the use of multiple imputation ‒ can serve to inspire other researchers on how to manage missing data.

## 1 Experimental design, materials, and methods

Full descriptions of the experimental design, materials, and methods can be found at the primary article [1].

### 1.1 Social-evaluative threat (SET) manipulation

**Fig. 1:**
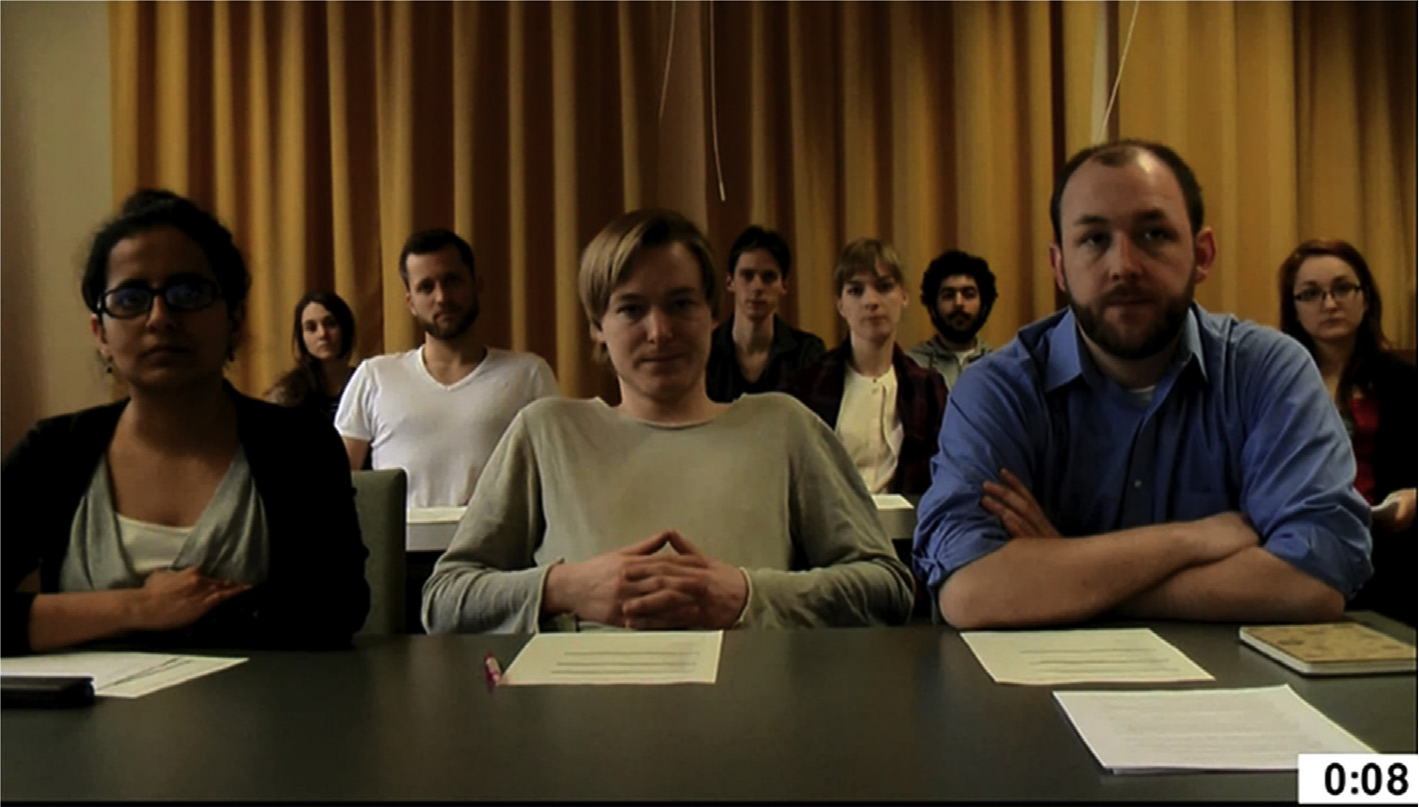
Screenshot of video audience and the timer (lower right corner).

Social-evaluative threat was induced using a public speaking task. Fig. 1 shows a screenshot of the video audience (with permission).

### 1.2 Assessments and measures

#### 1.2.1 Traits

We used self-report questionnaires to measure extraversion and neuroticism (Big Five Aspects Scale using twenty items each) [3], as well as related traits such as: BIS-BAS sensitivity (behavioral inhibition and approach scales; using seven items for BIS and twelve items for BAS) [4], social anxiety (Liebowitz Social Anxiety Scale; using 48 items) [5], self-esteem (Rosenberg Self-Esteem Scale; using ten items) [6], need to belong (Need to Belong scale; using ten items) [7,8], rumination (Post-event Rumination Questionnaire; using eight items for positive rumination (excl. items #4, 12, 20) and thirteen items for negative rumination (excl. items #5, 7, 15)) [9,10], and masculinity and femininity (Multifaceted Gender-Related Attributes Survey; using three items each) [11]. Additionally, English language competence (Cambridge online test using 25 items; www.cambridgeenglish.org/test-your-english/general-english/) was measured as a confounding variable.

Based on relevance in the literature and our hypotheses, only the extraversion and neuroticism traits were selected to be featured in the regression models and in the primary article.

#### 1.2.2 Self-reported appraisals

Resource and demand appraisals (stage one of the stress response [12]) were both assessed with single questions. Demand appraisal was measured with: “How demanding do you expect the upcoming task to be?” and resource appraisal with: “How able are you to cope with the upcoming task?”. A continuous composite measure of resources and demands was calculated, by subtracting demands from resources, yielding positive values in case of higher resources than demands (challenge) and negative values in case of higher demands than re-sources (threat).

#### 1.2.3 Cardiovascular physiology

Cardiovascular physiology was recorded to measure the following indices of stage two of the stress response: heart rate (HR), mean blood pressure (BP), pre-ejection period (PEP), and respiratory sinus arrhythmia (RSA), as well as respiratory rate (RR) as a covariate in RSA analyses [13]. Electrocardiography (ECG), impedance cardiography (ICG), and respiration were recorded continuously, while systolic and diastolic BP was measured repeatedly. The ECG and ICG signals were analyzed using ANSLAB [14], according to standard analysis protocols. Mean blood pressure was calculated using the formula: 2/3 diastolic +1/3 systolic [16].

Additional information is provided for ICG measures that were not discussed in the primary article but are included in the open dataset: cardiac output, total peripheral resistance, and threat-challenge index. Cardiac output (CO in liters per minute) was calculated by multiplying heart rate with stroke volume (as estimated in ANSLAB [14] using the Kubicek formula [15]). Total peripheral resistance (TPR in dyne-seconds * cm^−5^) was computed by dividing mean blood pressure by CO and multiplying that value by 80 [17]. A threat-challenge index for each time point was calculated by subtracting z-transformed-values of TPR from CO [18,19]. Thus, higher values on the TCI indicate a stronger challenge motivational state whereas lower values on the TCI indicate a stronger threat motivational state.

#### 1.2.4 Self-reported affective and motivational states

Affective and motivational responses (stage three of the stress response) were measured using state anxiety and state approach motivation, respectively [10,20,21]. State anxiety was measured with the single question: “How anxious do you feel right now?”, and state approach motivation was measured with the single question: “How much are you looking forward to the next part of the study?”.

#### 1.2.5 Endocrine physiology

In order to assess free salivary cortisol (stage four of the stress response), seven saliva samples were collected throughout the experiment and frozen. Analysis was performed using ELISA (DeMediTec Diagnostics, Kiel, Germany) by using two duplicate measures for each saliva sample to increase reliability, and samples with intra-assay coefficients of variability above 25% were repeated.

### 1.3 Statistical analyses

#### 1.3.1 Outlier detection

Outliers were detected based on significant values on the Grubbs test [22]. This statistic tests the deviation from the sample mean of the largest and smallest observation of a given variable. This test was applied over all variables (with Bonferroni-correction), and repeated until no significant outliers were present (i.e., after one round). Two outliers were excluded in these steps. Subsequently, the regression models using complete observations were tested for outliers in the Studentized residuals of each linear model (with Bonferroni-correction), based on the mean-shift outlier test [23]. One outlier was excluded in this step, resulting in three outlier participants in total.

#### 1.3.2 Missing data management

A description of all missing observations and outliers per variable can be found in Table 1. Variables that did not contain any missing data or outliers are not included in Table 1 (Neuroticism, Extraversion, Resource-demand appraisal, State anxiety 1 through 4, ΔState anxiety, State approach motivation 1 through 4, ΔState approach motivation, Mean blood pressure 1 through 8, ΔMean blood pressure, Cortisol 2 through 7).

**Table 1:** Missing observations and outliers per variable.

Variable	Number of missing observations	Number of outliers
State anxiety 5	7	0
State anxiety 6	7	0
State anxiety 7	7	0
State anxiety 8	7	0
State approach motivation 5	7	0
State approach motivation 6	7	0
State approach motivation 7	7	0
State approach motivation 8	7	0
HR 1	1	0
HR 2	1	0
HR 3	2	0
HR 4	3	1
HR 5	2	0
HR 6	2	0
HR 7	4	0
HR 8	4	0
ΔHR	5	0
PEP 1	6	0
PEP 2	6	0
PEP 3	6	0
PEP 4	6	0
PEP 5	7	0
PEP 6	6	0
PEP 7	8	0
PEP 8	8	0
ΔPEP	9	0
RSA 1	1	1
RSA 2	1	1
RSA 3	2	1
RSA 4	3	1
RSA 5	2	1
RSA 6	2	1
RSA 7	4	1
RSA 8	4	1
ΔRSA	4	1
RR 1	5	0
RR 2	4	0
RR 3	3	0
RR 4	4	0
RR 5	3	0
RR 6	3	0
RR 7	5	0
RR 8	5	0
ΔRR	7	0
Cortisol 1	0	1
ΔCortisol	0	1

*Note.* HR = heart rate; PEP = pre-ejection period; RSA = respiratory sinus arrhythmia; RR = respiratory rate; Δ = individual reactivity.

#### 1.3.3 Multiple imputation of missing data

Since twenty-four participants had some missing data points due to excessive noise, temporary sensor malfunction, or loose contacts, and another four participants had excluded outlier data points (see section *Outlier detection*), there were only thirty-eight complete observations in the dataset out of sixty-seven. To avoid the loss of 43.3% of our participants in the analyses, we multiply imputed the missing data using chained equations using the MICE package [24]; a “state of the art” missing data method.

The imputation model did not contain all possible variables, considering the large number of variables in the dataset. Instead, only relevant variables were included for all variables to be imputed (as is recommended: Buuren and Groothuis-Oudshoorn, 2011): sex, age, trait extraversion, trait neuroticism, resource-demand appraisal, and all reactivity variables, as well as the other time points of the same measure; resulting in twenty to twenty-one predictors per variable. This is specified in the predictorMatrixAdj.xlsx file [2]. (For example, HR 1 was predicted by: sex, age, trait extraversion, trait neuroticism, resource-demand appraisal, reactivity variables of: state anxiety, state approach motivation, mean BP, PEP, RSA, RR, and cortisol, as well as the other HR time points: 2, 3, 4, 5, 6, 7, and 8.) Reactivity variables were passively imputed, based on a given formula to compute individual peak minus baseline (Δ; see *SET reactivity* section in primary article).

Forty-four datasets were imputed, based on the rule of thumb that at least as many datasets need to be imputed as the percentage of incomplete cases [25]. Missing values were imputed by predictive mean matching, since in this method imputations are restricted to the observed values [24]. Two-hundred iterations were allowed to reach convergence.

Plausibility of imputed variables was assessed by comparing them to complete observations using boxplots, strip plots, and density plots, and summary statistics. All subsequent analyses were performed for each of the imputed datasets and the resulting estimates were pooled according to Rubin's rules [26].

#### 1.3.4 SET reactivity

SET responses were computed with a reactivity measure of individual peak minus baseline [27], henceforth identified as Δ. The peak represents the individual maximum or minimum value (depending on the measure) during or right after SET (i.e., either early or late anticipation, or early or late first recovery). Additionally, we calculated the area under the curve (AUC with respect to the increase [28]) for the cortisol response, which were strongly correlated, r = 0.93, p < .001.

## 2 Data

Raw and analyzed data can be accessed via Mendeley data [2].

In this section, we will report the correlation coefficients of associations between trait predictors (extraversion, neuroticism) and baseline state measures, as well as between different stress response measures. Additionally, scatterplots of significant associations between baseline states and traits and stress responses are provided. Finally, we report on the sex differences in the timing of the peak stress response reactivity.

### 2.1 Associations between stress response systems

Correlations between stress response systems were computed using Pearson correlations, in particular: between trait predictors (extraversion, neuroticism) and baseline state measures (Table 2), between different stress response measures (Table 2), between trait predictors (extraversion, neuroticism) and baseline state measures per sex (Table 3), and between different stress response measures per sex (Table 3).

**Table 2:** Correlations between trait predictors (extraversion, neuroticism), baseline state measures, and different stress response measures.

Baseline states and traits		Extraversion	Neuroticism	Baseline PEP	Baseline RSA	Baseline state approach motivation	Baseline state anxiety	Baseline Cortisol	Resource-demand appraisal	ΔPEP	ΔRSA	ΔState approach motivation	ΔState anxiety	ΔCortisol
Extraversion	*r*													
	*p*													
Neuroticism	*r*	−.34												
	*p*	.005												
Baseline PEP	*r*	−.14	.26											
	*p*	.266	.038											
Baseline RSA	*r*	−.15	.05	.02										
	*p*	.241	.683	.867										
Baseline state approach motivation	*r*	.09	.08	.04	−.01									
	*p*	.484	.545	.779	.927									
Baseline state anxiety	*r*	.09	.01	−.07	.25	.09								
	*p*	.464	.965	.557	.040	.479								
Baseline Cortisol	*r*	.22	−.11	−**.39**	−.23	−.09	.06							
	*p*	.076	.372	.001*	.066	.491	.629							
Resource-demand appraisal	*r*	−.04	.09	.06	.05	.12	−.17	−.16						
	*p*	.734	.470	.613	.709	.347	.162	.210						
ΔPEP	*r*	.20	.25	−.30	−.18	.05	−.07	.09	−.13					
	*p*	.125	.043	.017	.208	.688	.603	.504	.313					
ΔRSA	*r*	.05	.13	.18	−**.49**	.10	−.18	.09	−.05	.23				
	*p*	.697	.315	.149	<.001**	.415	.165	.465	.705	.094				
ΔState approach motivation	*r*	−.12	.10	−.02	−.19	−**.36**	−.15	−.03	.13	−.04	−.05			
	*p*	.336	.416	.894	.124	.003*	.220	.810	.302	.785	.726			
ΔState anxiety	*r*	−.11	−.04	.21	−.17	<.01	−**.52**	.03	.16	−.08	.22	−.26		
	*p*	.363	.777	.092	.174	.993	<.001***	.790	.197	.554	.075	.035		
ΔCortisol	*r*	−.13	−**.36**	−.05	−.12	−.20	−.23	−.07	.02	−**.43**	−.16	.14	.11	
	*p*	.290	.003*	.712	.332	.100	.087	.572	.878	<.001*	.232	.268	.372	

*Note.* Significant correlations are shown in bold (FDR-corrected *p* < .05); ** = significant at α = 0.01 after FDR correction; * = significant at α = 0.05 after FDR correction. PEP = pre-ejection period, RSA = respiratory sinus arrhythmia; Δ = individual reactivity.

**Table 3:** Correlations between trait predictors (extraversion, neuroticism), baseline state measures, and different stress response measures per sex (women above, men below diagonal).

Baseline states and traits		Extraversion	Neuroticism	Baseline PEP	Baseline RSA	Baseline state approach motivation	Baseline state anxiety	Baseline Cortisol	Resource-demand appraisal	ΔPEP	ΔRSA	ΔState approach motivation	ΔState anxiety	ΔCortisol
Extraversion	*r*		−.38	−.34	−.18	.01	.15	.38	−.03	.43	−.03	−.12	−.15	−.25
	*p*		.035	.065	.347	.951	.439	.040	.857	.024	.864	.539	.432	.201
Neuroticism	*r*	−**.19**		.34	−.09	.17	−.12	−.14	.04	.01	.42	−.08	.30	−.26
	*p*	**.252**		.068	.637	.370	.532	.463	.816	.950	.025	.689	.109	.162
Baseline PEP	*r*	**.14**	**.17**		.02	−.27	−.18	−.40	.03	−.34	.15	.18	.16	.06
	*p*	**.414**	**.326**		.915	.150	.344	.027	.873	.070	.431	.356	.390	.773
Baseline RSA	*r*	−**.09**	**.09**	**.01**		−.03	.16	−.19	.22	−.25	−.49	−.27	−.16	.12
	*p*	**.593**	**.583**	**.947**		.864	.412	.335	.261	.235	.011	.164	.395	.553
Baseline state approach motivation	*r*	**.19**	−**.02**	**.30**	−**.01**		−.03	−.06	.13	.23	.05	−.39	−.06	−.22
	*p*	**.266**	**.924**	**.083**	**.976**		.859	.768	.502	.276	.783	.032	.758	.251
Baseline state anxiety	*r*	**.07**	**.05**	**.03**	**.32**	**.18**		.09	−.11	−.05	−.07	−.17	−.51	−.20
	*p*	**.686**	**.776**	**.865**	**.051**	**.276**		.639	.564	.797	.710	.363	.004	.354
Baseline Cortisol	*r*	**.05**	−**.14**	−**.38**	−**.28**	−**.12**	**.02**		−.32	.02	.08	−.10	.08	−.10
	*p*	**.762**	**.414**	**.023**	**.093**	**.492**	**.912**		.086	.923	.677	.612	.674	.638
Resource-demand appraisal	*r*	−**.06**	**.16**	**.10**	−**.07**	**.11**	−**.23**	**<.01**		−.18	−.15	.02	.16	−.22
	*p*	**.705**	**.353**	**.566**	**.677**	**.514**	**.174**	**.984**		.342	.458	.914	.403	.251
ΔPEP	*r*	−**.01**	**.44**	−**.27**	−**.14**	−**.08**	−**.09**	**.15**	−**.08**		.39	−.18	−.07	−.35
	*p*	**.951**	**.006**	**.120**	**.466**	**.664**	**.596**	**.415**	**.645**		.052	.387	.725	.067
ΔRSA	*r*	**.13**	−**.10**	**.25**	−**.53**	**.18**	−**.31**	**.12**	**.06**	**.06**		.04	.15	−.41
	*p*	**.465**	**.567**	**.160**	**.002**	**.309**	**.069**	**.476**	**.708**	**.735**		.846	.442	.030
ΔState approach motivation	*r*	−**.09**	**.22**	−**.26**	−**.15**	−**.35**	−**.15**	**.04**	**.25**	**.10**	−**.15**		−.15	.16
	*p*	**.608**	**.184**	**.129**	**.376**	**.031**	**.384**	**.809**	**.144**	**.579**	**.382**		.441	.415
ΔState anxiety	*r*	−**.07**	−**.29**	**.24**	−**.19**	**.03**	−**.57**	−**.01**	**.17**	−**.09**	**.33**	−**.37**		.11
	*p*	**.701**	**.079**	**.156**	**.275**	**.883**	<.001*	**.971**	**.326**	**.614**	**.047**	**.023**		.565
ΔCortisol	*r*	−**.10**	−**.37**	−**.12**	−**.27**	−**.18**	−**.23**	−**.04**	**.20**	−**.50**	**.10**	**.16**	**.14**	
	*p*	**.565**	**.025**	**.473**	**.107**	**.277**	**.165**	**.809**	**.236**	**.003**	**.590**	**.352**	**.416**	

*Note.* Men are shown underneath the diagonal in bold; women are shown above the diagonal. * = significant at α = 0.05 after FDR correction. PEP = pre-ejection period, RSA = respiratory sinus arrhythmia; Δ = individual reactivity.

For all analyses, alpha was set at .05, and false-discovery rate (FDR) correction was performed to correct for multiple comparisons. Uncorrected *p*-values are reported for transparency, with FDR-corrected significance indicated by superscript symbols.

When combining men and women, the only significant FDR-corrected correlations were those between PEP and cortisol, both for baseline and reactivity indices ‒ indicating more sympathetic nervous system (SNS) activity with more hypothalamus-pituitary-adrenal (HPA) axis activity ‒ as well as between baseline and reactivity for RSA, state approach motivation, and state anxiety, and between neuroticism and Δcortisol. No correlations for each sex separately were significant after FDR-correction.

### 2.2 Scatterplots of significant associations

**Fig. 2:**
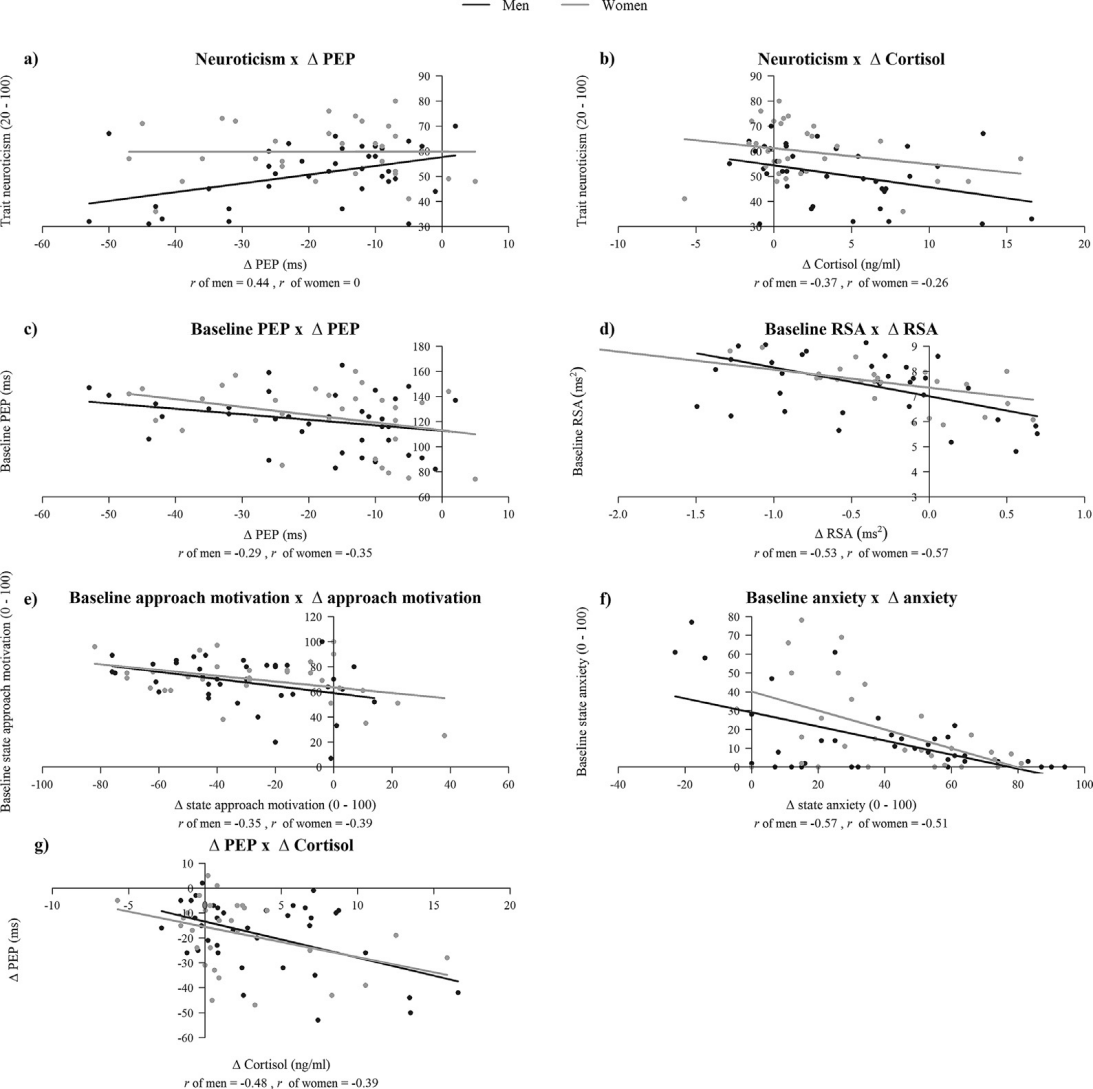
Scatterplots of significant associations between trait predictors, baseline state measures, and different stress response measures per sex: a) trait neuroticism with ΔPEP, b) trait neuroticism with Δcortisol, c) baseline PEP with ΔPEP, d) baseline RSA with ΔRSA, e) baseline state approach motivation with Δstate approach motivation, f) baseline state anxiety with Δstate anxiety, and g) ΔPEP with Δcortisol.

Scatterplots of significant regression associations between trait predictors (extraversion, neuroticism), baseline state measures, and different stress response measures per sex are shown in Fig. 2. The first imputed dataset (see section: *Multiple imputation of missing data*) was used for illustration purposes (N = 67).

### 2.3 Peak timing

Sex differences in the timing of the peak reactivity were assessed using two-sample *t*-tests (variances not assumed equal). Sex differences in RSA were tested using a linear regression with RR as covariate. The regression coefficients were then converted into *t*-values. To provide confirming evidence of the null hypotheses, Bayes factors were calculated from *t*-values using the BayesFactor package [29] with default non-informative priors. Alpha was set at .05, and FDR correction was performed to correct for multiple comparisons. Uncorrected *p*-values are reported for transparency, with FDR-corrected significance indicated by superscript symbols.

Results are shown in Table 4. Peak time of the decrease in RSA (corrected for RR) was earlier in women than men and peak time of the decrease in PEP was comparable between men and women. Peak time reactivity of state anxiety, state approach motivation, mean BP, heart rate, RSA (uncorrected for RR), RR, and cortisol did not differ significantly between men and women, although based on Bayes factors there was inconclusive evidence to support neither equal nor different group means.

**Table 4:** Sex differences in time of peak reactivity.

SET reactivity	Sex	Mean	*SD*	*t* (df)	*p*	BF
ΔState anxiety	Male	13.81	3.9	1.06 (63)	.295	0.40 ^inc.^
	Female	12.87	3.4			
ΔState approach motivation	Male	12.51	3.2	1.04 (63)	.303	0.40 ^inc.^
	Female	11.80	2.4			
ΔMean BP	Male	20.35	8.5	0.94 (63)	.352	0.37 ^inc.^
	Female	22.17	7.3			
ΔHeart rate	Male	6.52	1.2	2.33 (56)	.024	2.40 ^inc.^
	Female	5.80	1.2			
ΔPEP	Male	8.21	5.7	0.19 (49)	.852	0.26^H0^
	Female	8.52	6.8			
ΔRSA	Male	12.52	9.4	1.27 (58)	.211	0.50 ^inc.^
	Female	9.73	8.0			
ΔRSA (corrected for RR)				4.51 (57)	<.001***	2.62*10^2H1^
		
ΔRR	Male	14.98	9.8	1.04 (57)	.302	0.40 ^inc.^
	Female	12.43	9.4			
ΔCortisol	Male	33.73	6.0	1.58 (48)	.120	0.72 ^inc.^
	Female	30.80	8.5			

*Note.* Mean peak time in minutes after onset of SET manipulation (duration of 18 minutes). *SD* = standard deviation; BF = Bayes factor; BP = blood pressure; PEP = pre-ejection period; RSA = respiratory sinus arrhythmia; RR = respiratory rate; Δ = individual reactivity. *** = significant at α = .001 after FDR correction; H0 = evidence in support of equal group estimates; H1 = evidence in support of different group means; inc. = inconclusive evidence in support of neither equal nor different group means.
